# Supplementary material for: Microsatellite Status Detection in Gastrointestinal Cancers: PCR/NGS Is Mandatory in Negative/Patchy MMR Immunohistochemistry
Source: Cancers (Basel). 2022 Apr 28;14(9):2204. doi: 10.3390/cancers14092204 (PMC9102010; doi:10.3390/cancers14092204)
Supplement: Supplementary file 1 [file cancers-14-02204-s001.zip › cancers-1591809-supplementary.pdf]

Supplementary material

Microsatellite Status Detection in Gastrointestinal Cancers: PCR/NGS is Mandatory in Negative/Patchy MMR Immunohistochemistry

Federica Zito Marino, Martina Amato, Andrea Ronchi, Iacopo Panarese, Francesca Ferraraccio, Ferdinando De Vita, Giuseppe Tirino, Erika Martinelli, Teresa Troiani, Gaetano Facchini, Felice Pirozzi, Michele Perrotta, Pasquale Incoronato, Raffaele Addeo, Francesco Selvaggi, Francesco Saverio Lucido, Michele Caraglia, Giovanni Savarese, Roberto Sirica, Marika Casillo, Eva Lieto, Annamaria Auricchio, Francesca Cardella, Ludovico Docimo, Gennaro Galizia and Renato Franco

Supplementary data

Table S1. Comparison of MMR/IHC, MSI/RT-PCR and PD-L1 expression.

	CASES	IHC RESULTS				RT-PCR RESULTS	PD-L1 EXPRESSION
		MLH1	PMS2	MSH2	MSH6		
dMMR	1	-	-	+	+	MSI-H	NEG
	2	-	-	+	+	MSS	80%
	3	-	-	+	+	MSI-H	NEG
	4	-	-	+	+	MSI-H	NEG
	5	-	-	+	+	MSI-H	NEG
	6	-	-	+	+	MSI-H	NEG
	7	-	-	+	+	MSI-H	>90%
	8	-	-	+	+	MSI-H	NEG
	9	-	-	+	+	MSI-H	NEG
	10	-	-	+	+	MSI-H	NEG
	11	-	-	+	+	MSI-H	NEG
	12	-	-	+	+	MSS	NEG
	13	-	-	+	+	MSI-H	NEG
	14	-	-	+	+	MSI-H	NEG
	15	+	+	-	-	MSI-H	NEG
LoMMR	16	+	-	+	+	MSS	NEG
	17	+	-	+	+	MSI-H	30%

	18	+	-	+	+	MSI-H	NEG
	19	+	-	+	+	MSI-H	50%
	20	+	+	+	-	MSI-H	NEG
	21	+	-	+	+	MSS	NEG
	22	+	-	+	+	MSI-H	NEG
	23	+	-	+	+	MSI-H	10%
	24	+	-	+	+	MSS	NEG
	25	+	-	+	+	MSI-H	2%
	26	+	+	pa	+	MSS	NEG
	27	pa	pa	+	+	MSS	NEG
	28	+	pa	+	pa	MSS	NEG
	29	+	pa	+	+	MSS	NEG
	30	+	pa	+	+	MSS	NEG
	31	pa	pa	+	+	MSS	NEG
	32	pa	pa	+	+	MSS	NEG
	33	+	pa	+	+	MSS	NEG
	34	+	pa	+	+	MSS	NEG
	35	+	pa	+	+	MSS	NEG
	36	+	pa	+	+	MSS	NEG
	37	+	pa	+	+	MSS	NEG
	38	+	pa	+	+	MSS	NEG
	39	+	pa	+	+	MSS	NEG
	40	+	pa	+	+	MSI-L	NEG
**paMMR**	41	+	pa	+	+	MSI-H	NEG
	42	+	pa	+	+	MSS	NEG
	43	+	+	pa	pa	MSI-L	NEG
	44	pa	pa	+	+	MSI-H	NEG
	45	pa	pa	+	+	MSS	NEG
	46	+	pa	+	+	MSS	NEG
	47	+	pa	+	+	MSS	NEG
	48	+	pa	+	+	MSS	NEG
	49	+	pa	+	+	MSS	NEG
	50	+	pa	+	+	MSS	NEG
	51	+	pa	+	+	MSS	NEG
	52	+	pa	+	+	MSI-H	NEG
	53	+	pa	+	+	MSS	NEG
	54	+	pa	+	+	MSI-L	NEG
	55	pa	pa	+	+	MSS	1%
	56	+	pa	+	+	MSS	NEG
	57	pa	pa	+	-	MSS	NEG
	58	pa	-	+	+	MSS	NEG
**Lo-paMMR**	59	pa	-	+	+	MSI-H	NEG
	60	pa	-	+	+	MSI-H	NEG
	61	pa	pa	pa	-	MSI-H	NEG

Table S2. Comparison between IHC and RT-PCR in CRC patients.

Case	IHC				RT-PCR								Results MMR-IHC/MSI-PCR
	MLH1	PMS2	MSH2	MSH6	BAT25	BAT26	NR21	NR22	NR24	NR27	CAT25	MONO27	
1	+	+	+	-	I	I	I	S	S	S	I	I	loMMR/MSI-H
2	-	-	+	+	I	I	S	S	I	I	I	I	dMMR/MSI-H
3	+	-	+	+	I	I	I	S	S	S	I	I	loMMR/MSI-H
4	-	-	+	+	I	I	I	S	S	I	I	S	dMMR/MSI-H
5	-	-	+	+	S	S	S	S	S	S	S	S	dMMR/MSS
6	-	-	+	+	I	I	I	I	I	I	I	I	dMMR/MSI-H
7	-	-	+	+	I	I	I	S	S	I	I	I	dMMR/MSI-H
8	-	-	+	+	I	I	I	I	I	I	I	I	dMMR/MSI-H
9	-	-	+	+	I	I	I	I	S	I	I	S	dMMR/MSI-H
10	-	-	+	+	I	I	I	I	I	I	I	I	dMMR/MSI-H
11	+	-	+	+	S	S	S	S	S	S	S	S	loMMR/MSS
12	-	-	+	+	I	I	I	I	I	I	I	I	dMMR/MSI-H
13	-	-	+	+	I	I	I	I	I	I	I	I	dMMR/MSI-H
14	-	-	+	+	I	I	I	S	S	I	S	S	dMMR/MSI-H
15	+	-	+	+	I	I	I	I	I	I	I	I	loMMR/MSI-H
16	+	-	+	+	I	I	I	I	I	I	I	I	loMMR/MSI-H
17	-	-	+	+	S	S	S	S	S	S	S	S	dMMR/MSS
18	+	+	+	-	I	S	I	S	S	I	I	S	loMMR/MSI-H
19	+	-	+	+	S	S	S	S	S	S	S	S	lo/MMR/MSS
20	+	-	+	+	I	I	I	I	I	I	I	S	loMMR/MSI-H
21	-	-	+	+	S	I	I	S	I	I	I	S	dMMR/MSI-H
22	+	-	+	+	I	I	I	I	I	I	I	I	loMMR/MSI-H
23	+	+	-	-	I	I	S	S	I	I	I	S	dMMR/MSI-H
24	-	-	+	+	I	I	I	I	I	I	I	I	dMMR/MSI-H
25	+	-	+	+	S	S	S	S	S	S	S	S	loMMR/MSS
26	+	+	pa	+	S	S	S	S	S	S	S	S	paMMR/MSS
27	pa	pa	+	+	S	S	S	S	S	S	S	S	paMMR/MSS
28	+	pa	+	P	S	S	S	S	S	S	S	S	paMMR/MSS
29	+	pa	+	+	S	S	S	S	S	S	S	S	paMMR/MSS
30	+	pa	+	+	S	S	S	S	S	S	S	S	paMMR/MSS

**31**	pa	pa	+	+	S	S	S	S	S	S	S	S	**paMMR/MSS**
**32**	pa	pa	+	+	S	S	S	S	S	S	S	S	**paMMR/MSS**
**33**	+	pa	+	+	S	S	S	S	S	S	S	S	**paMMR/MSS**
**34**	+	pa	+	+	S	S	S	S	S	S	S	S	**paMMR/MSS**
**35**	+	pa	+	+	S	S	S	S	S	S	S	S	**paMMR/MSS**
**36**	+	pa	+	+	S	S	S	S	S	S	S	S	**paMMR/MSS**
**37**	+	pa	+	+	S	S	S	S	S	S	S	S	**paMMR/MSS**
**38**	+	pa	+	+	S	S	S	S	S	S	S	S	**paMMR/MSS**
**39**	+	pa	+	+	S	S	S	S	S	S	S	S	**paMMR/MSS**
**40**	+	pa	+	+	I	S	S	S	S	S	S	S	**paMMR/MSI-L**
**41**	+	pa	+	+	I	I	I	S	I	I	I	I	**paMMR/MSI-H**
**42**	+	pa	+	+	S	S	S	S	S	S	S	S	**paMMR/MSS**
**43**	+	+	pa	pa	S	S	I	S	S	S	S	S	**paMMR/MSI-L**
**44**	pa	pa	+	+	S	I	S	I	I	I	I	I	**paMMR/MSI-H**
**45**	pa	pa	+	+	S	S	S	S	S	S	S	S	**paMMR/MSS**
**46**	+	pa	+	+	S	S	S	S	S	S	S	S	**paMMR/MSS**
**47**	+	pa	+	+	S	S	S	S	S	S	S	S	**paMMR/MSS**
**48**	+	pa	+	+	S	S	S	S	S	S	S	S	**paMMR/MSS**
**49**	+	pa	+	+	S	S	S	S	S	S	S	S	**paMMR/MSS**
**50**	+	pa	+	+	S	S	S	S	S	S	S	S	**paMMR/MSS**
**51**	+	pa	+	+	S	S	S	S	S	S	S	S	**paMMR/MSS**
**52**	+	pa	+	+	S	I	I	S	I	I	I	I	**paMMR/MSI-H**
**53**	+	pa	+	+	S	S	S	S	S	S	S	S	**paMMR/MSS**
**54**	+	pa	+	+	S	S	I	S	S	S	S	S	**paMMR/MSI-L**
**55**	pa	pa	+	+	S	S	S	S	S	S	S	S	**paMMR/MSS**
**56**	+	pa	+	+	S	S	S	S	S	S	S	S	**paMMR/MSS**
**57**	pa	pa	+	-	S	S	S	S	S	S	S	S	**lo-paMMR/MSS**
**58**	pa	-	+	+	S	S	S	S	S	S	S	S	**lo-paMMR/MSS**
**59**	pa	-	+	+	I	I	I	I	I	I	I	I	**lo-paMMR/MSI-H**
**60**	pa	-	+	+	I	I	I	I	I	I	I	I	**lo-paMMR/MSI-H**
**61**	pa	-	+	+	I	S	I	S	S	I	I	S	**lo-paMMR/MSI-H**

MMR: Mismatch Repair; MSI: Microsatellite Instability; dMMR: deficient Mismatch Repair; IHC: Immunohistochemistry; pa: patchy; paMMR: patchy Mismatch Repair; loMMR: loss one Mismatch Repair; lo-paMMR: loss one and patchy Mismatch Repair; MLH1: MutL Homolog human 1; MSH2: MutS Homolog human 2; MSH6: MutS Homolog human 6; PMS2: Postmeiotic Segregation Increased 2; MSI-H: Microsatellite Instability-High; MSS: Microsatellite Stability; MSI-L: Microsatellite Instability-Low.

Table S3. Comparison between IHC, RT-PCR and NGS results in CRC case.

Case	IHC results				RT-PCR results	NGS results
	**MLH1**	**PMS2**	MSH2	MSH6		
**1**	-	-	+	+	MSS	MSS
**2**	-	-	+	+	MSS	MSS
**3**	+	+	-	-	MSI-H	MSI-H
**4**	+	-	+	+	MSI-H	MSI-H
**5**	+	-	+	+	MSI-H	MSI-H
**6**	+	-	+	+	MSI-H	MSI-H
**7**	+	-	+	+	MSI-H	MSI-H
**8**	+	-	+	+	MSI-H	MSI-H
**9**	+	-	+	+	MSI-H	MSI-H
**10**	+	+	+	-	MSI-H	MSI-H
**11**	+	+	+	-	MSI-H	MSI-H
**12**	pa	-	+	+	MSI-H	MSI-H
**13**	pa	-	+	+	MSI-H	MSI-H
**14**	pa	-	+	+	MSS	MSS
**15**	pa	pa	+	+	MSI-H	MSI-H
**16**	pa	pa	+	+	MSS	MSS
**17**	+	+	pa	pa	MSI-L	MSS
**18**	+	pa	+	+	MSI-L	MSS
**19**	+	pa	+	+	MSI-L	MSS
**20**	+	pa	+	+	MSS	MSS
**21**	+	pa	+	+	MSS	MSS
**22**	+	pa	+	+	MSS	MSS
**23**	+	pa	+	+	MSS	MSS
**24**	+	pa	+	+	MSI-H	MSI-H
**25**	+	pa	+	+	MSI-H	MSI-H

IHC: Immunohistochemistry; NGS: Next-Generation Sequencing; MLH1: MutL Homolog human 1; MSH2: MutS Homolog human 2; MSH6: MutS homolog human 6; PMS2: Postmeiotic Segregation Increased 2; pa: patchy; MSI-H: Microsatellite Instability-High; MSS: Microsatellite Stability; MSI-L: Microsatellite Instability-Low.

Table S4. Comparison between IHC and RT-PCR in GC patients.

Cases	IHC				RT-PCR								Results
	MLH1	PMS2	MSH2	MSH6	BAT25	BAT26	NR21	NR22	NR24	NR27	CAT25	MONO27	MMR-IHC/MSI-PCR
**1**	+	pa	+	+	S	S	S	S	S	S	S	S	paMMR/MSS
**2**	pa	pa	+	+	S	S	S	S	S	S	S	S	paMMR/MSS
**3**	+	pa	+	+	S	S	S	S	S	S	S	S	paMMR/MSS
**4**	+	pa	+	+	S	S	I	S	S	S	S	S	paMMR/MSI-L
**5**	pa	pa	+	+	S	S	S	S	S	S	S	S	paMMR/MSS
**6**	-	-	+	+	I	I	I	I	I	I	I	I	dMMR/MSI-H
**7**	-	-	+	+	I	I	S	I	I	I	I	I	dMMR/MSI-H
**8**	-	-	+	+	I	I	S	S	S	I	I	I	dMMR/MSI-H
**9**	-	-	+	+	I	I	I	I	I	I	I	I	dMMR/MSI-H
**10**	-	-	+	+	I	I	S	S	S	I	I	S	dMMR/MSI-H
**11**	-	-	+	+	I	I	S	S	S	I	S	I	dMMR/MSI-H
**12**	+	-	+	+	S	S	S	S	S	S	S	S	loMMR/MSS
**13**	-	-	+	+	I	I	I	I	I	I	I	I	dMMR/MSI-H
**14**	-	-	+	+	S	I	S	S	S	I	I	I	dMMR/MSI-H
**15**	-	-	+	+	I	I	I	I	I	I	I	I	dMMR/MSI-H
**16**	-	-	+	+	I	I	I	S	I	I	I	I	dMMR/MSI-H
**17**	-	-	+	+	S	S	S	S	S	S	S	S	dMMR/MSS
**18**	-	-	+	+	I	I	I	I	I	I	I	I	dMMR/MSI-H
**19**	-	-	+	+	I	I	I	I	I	I	I	I	dMMR/MSI-H

MMR: Mismatch Repair; MSI: Microsatellite Instability; dMMR: deficient Mismatch Repair; IHC: immunohistochemistry; pa: patchy; paMMR: patchy Mismatch Repair; loMMR: loss one Mismatch Repair; lo-paMMR: loss one and patchy Mismatch Repair; MLH1: MutL Homolog human 1; MSH2: MutS Homolog human 2; MSH6: MutS Homolog human 6; PMS2: Postmeiotic Segregation Increased 2; MSI-H: Microsatellite instability-high; MSS: microsatellite stability; MSI-L: Microsatellite instability- low.

Table S5. Comparison between IHC, RT-PCR and NGS results in GC cases.

Case	IHC results				RT-PCR results	NGS results
	MLH1	PMS2	MSH2	MSH6		
1	-	-	+	+	MSI-H	N.C.
	-	-	+	+	MSI-H	N.C.
3	-	-	+	+	MSI-H	N.C.
4	-	-	+	+	MSI-H	MSI-H
5	-	-	+	+	MSI-H	MSI-H
6	-	-	+	+	MSI-H	MSI-H
7	pa	pa	+	+	MSS	N.C.

IHC: Immunohistochemistry; NGS: Next-Generation Sequencing; MLH1: MutL Homolog human 1; MSH2: MutS Homolog human 2; MSH6: MutS Homolog human 6; PMS2: Postmeiotic Segregation Increased 2; pa: patchy; MSI-H: Microsatellite instability-high; MSS: microsatellite stability; NC: Not Contributive results
